# β-Hydroxybutyrate precursor 1,3-butanediol modulates enteric pathogen susceptibility and Th17 responses via commensal bacteria

**DOI:** 10.1128/msystems.00324-26

**Published:** 2026-07-16

**Authors:** Wenxuan Dong, Chi Yan, Bethany Korwin-Mihavics, Kate Stack, Gillian Hughes, Aidan Schmidt, Kevin J. Schwartz, Gustavo Caballero-Flores, Margaret Alexander

**Affiliations:** 1 Department of Medical Microbiology & Immunology, University of Wisconsin—Madison 5228, Madison, Wisconsin, USA; Chinese Academy of Sciences, Shanghai, China

**Keywords:** microbiota, microbiota immune interactions, T helper (Th) 17 cells, β-hydroxybutyrate, *Citrobacter rodentium* infection, segmented filamentous bacteria

## Abstract

T helper 17 (Th17) cells are a critical T lymphocyte subset involved in mucosal immunity and host defense against enteric pathogens. Although ketogenic diets (KD) and the major ketone body β-hydroxybutyrate (BHB) reshape gut microbiota and suppress Th17 responses under defined diet conditions, it remains unclear whether elevation of BHB alone, independent of dietary macronutrient composition and systemic metabolic shifts, is sufficient to remodel Th17-inducing commensals and alter host susceptibility to enteric infection. Here, we used 1,3-butanediol (BD), a precursor metabolized to BHB independently of KD, to elevate systemic BHB levels in mice. BD treatment significantly reduced the frequency of ileal Th17 cells, as assessed by flow cytometry for Th17 markers IL-17A and RORγt. 16S rRNA gene sequencing revealed that BD altered gut microbial community structure, as indicated by beta-diversity analysis based on Bray–Curtis dissimilarity, and reduced Shannon diversity and evenness. Linear discriminant analysis effect size identified segmented filamentous bacteria (SFB) as significantly decreased in the ileum following BD treatment, and SFB abundance positively correlated with Th17 markers. Microbiota transplantation demonstrated that BD-shaped microbiota was sufficient to suppress Th17 responses in recipient mice, accompanied by reduced SFB abundance. In a *Citrobacter rodentium* infection model, BD treatment was associated with increased pathogen burden, and fecal *C. rodentium* levels were negatively correlated with SFB abundance. Together, these results support a model in which BD treatment, accompanied by elevated circulating BHB, reshapes the commensal microbiota, including reducing SFB levels, and is associated with dampened Th17 responses as well as increased susceptibility to enteric infection.

**IMPORTANCE:**

Diet is a key determinant of gut microbial composition and mucosal immune function, yet the microbial mechanisms linking diet-mediated metabolic changes to immune regulation remain incompletely understood. T helper 17 (Th17) cells play central roles in both protective mucosal immunity and inflammatory pathology, making them a critical target of immunometabolic regulation. In this study, we show that 1,3-butanediol (BD) treatment, which leads to elevated circulating β-hydroxybutyrate (BHB) independently of diet, is associated with suppression of intestinal Th17 responses, remodeling of the gut microbiota, and reduced levels of segmented filamentous bacteria (SFB). We further demonstrate that BHB-associated microbiota changes are linked to increased susceptibility to enteric infection. This work provides a mechanistic framework illustrating how metabolic state can influence host immunity through selective effects on commensal microbes. These findings inform future studies of microbiota-mediated immune regulation.

## OBSERVATION

Dietary intervention has emerged as a promising strategy for modulating immune responses in inflammatory diseases. T helper 17 (Th17) cells play context-dependent roles in immunity, contributing to host defense against enteric pathogens while also driving pathogenic inflammation in autoimmune diseases (1–3). As a result, precise regulation of Th17 responses has been proposed as a means to balance protective immunity and inflammatory pathology. High-fat, low-carbohydrate ketogenic diets (KDs) that shift host metabolism to ketogenesis can alleviate autoimmune phenotypes through suppression of Th17 cells, an effect that has been associated with alterations in the gut microbiota (4–6). However, KDs profoundly change macronutrient composition and host metabolism, making it difficult to disentangle the immunological effects of ketogenesis from those of dietary fat and carbohydrate restriction. β-hydroxybutyrate (BHB), the major circulating ketone body produced during ketogenesis, functions as a signaling metabolite with roles in gene regulation and immune modulation, including effects on inflammasome activation and innate lymphoid cells (7–9). KDs and BHB also enhance antiviral immunity by modulating T cell metabolism and function, improving disease outcomes and survival during infections (10, 11). However, whether BHB alone is sufficient to regulate intestinal Th17 responses independently of diet and the impact on enteric infection susceptibility remains unclear. To address this question, we used 1,3-butanediol (BD), a metabolic precursor that elevates systemic BHB levels without altering dietary macronutrient composition, to isolate the effects of BHB from those of diet (12). Using a combination of flow cytometry, 16S rRNA gene sequencing, microbiota transplantation, and *Citrobacter rodentium* infection models, we examined how BHB influences Th17 responses and host susceptibility to enteric infection.

### Isolated elevation of BHB suppresses intestinal Th17 responses and reshapes the gut microbiota

**Fig 1:**
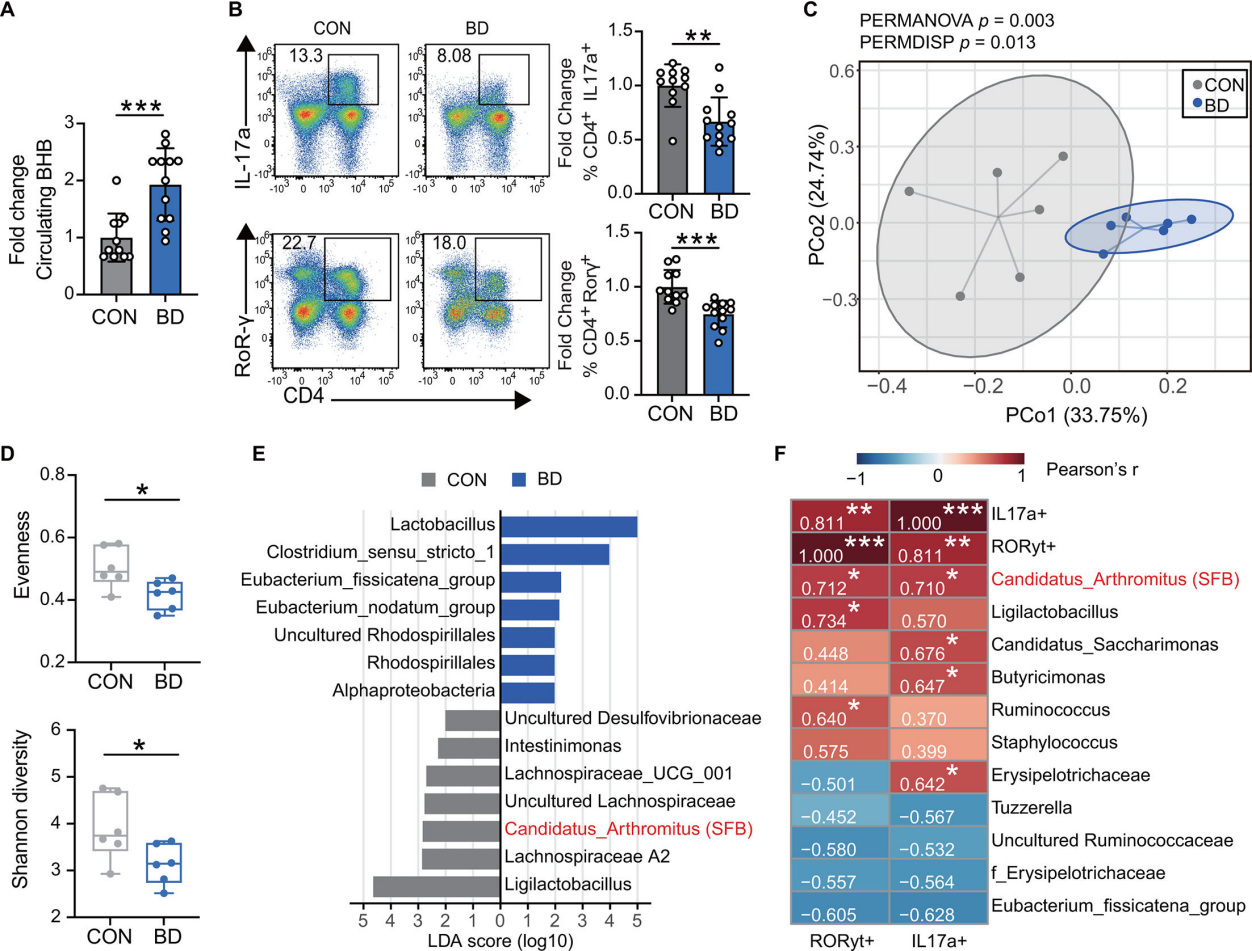
Elevated BHB decreases ileal Th17 levels and reshapes the microbiota. (**A**) Fold change in circulating β-hydroxybutyrate (BHB) levels in control (CON) and 1,3-butanediol (BD)-treated mice. Barplot shows mean ± standard deviation (SD). BD significantly increased circulating BHB compared to CON. Statistical analysis: unpaired two-tailed Welch’s *t* test. (**B**) Flow cytometry analysis of ileal lamina propria lymphocytes. Left, representative flow plots showing CD4^+^IL-17A^+^ and CD4^+^RORγt^+^ cells gated on live CD3^+^ populations. Right, quantification of Th17 frequency normalized to CON. Barplots show mean ± SD. Statistical analysis: unpaired two-tailed Welch’s *t* test. (**C**) Principal coordinates analysis (PCoA) based on Bray–Curtis dissimilarity showing differences in β-diversity between CON and BD groups. PERMANOVA and PERMDISP *p*-values are indicated. (**D**) Alpha diversity analysis of ileal microbiota. BD reduced community evenness (top) and Shannon diversity (bottom). Boxplots show the median and interquartile range (IQR), and whiskers represent the minimum and maximum values. Statistical analysis: Mann–Whitney *U* test. (**E**) Differentially abundant taxa identified by linear discriminant analysis effect size (LEfSe). Bars represent linear discriminant analysis (LDA) scores (log10 scale), and colors indicate taxa enriched in each group. (**F**) Pearson’s correlation heatmap between selected bacterial taxa (|correlation coefficient| > 0.5 and *p* < 0.1 for correlation with either RORγt^+^ or IL-17A^+^ CD4^+^ cells) and Th17 markers (IL-17A^+^ and RORγt^+^ CD4^+^ cells). Segmented filamentous bacteria (SFB; annotated as *Candidatus Arthromitus*) is highlighted in red. Numbers represent Pearson’s correlation coefficients. *P < 0.05, **P < 0.01, and ***P < 0.001.

To test whether BHB suppresses Th17 cells independently of diet, mice were supplemented with 20% BD in drinking water to elevate systemic BHB levels (12–14) (Fig. 1A). Compared to control mice (CON), BD-treated mice exhibited a significant reduction in ileal Th17 cell frequency, as defined by CD4^+^IL-17A^+^ and CD4^+^RORγt^+^ populations among total live CD3^+^ cells (Fig. 1B and Fig. S1). These results indicate that BD treatment, which leads to elevated systemic BHB in the absence of other dietary alterations, is sufficient to decrease intestinal Th17 responses.

Given the well-established role of the gut microbiota in Th17 cell responses, we next examined whether BD-mediated BHB production altered the intestinal microbial community. 16S rRNA gene sequencing revealed that BD significantly changed ileal microbiota composition compared to CON (Fig. 1C and Fig. S2). PERMANOVA revealed a significant difference in the community composition between groups, and PERMDISP indicated reduced beta-dispersion in the BD group, suggesting increased microbiome homogeneity. BD was also associated with reduced alpha diversity, including lower Shannon diversity and evenness (Fig. 1D). Previous studies have shown reduced Th17 cells in germ-free and antibiotic-treated mice, and specific members of the microbiota are known to elevate Th17 levels (15, 16), raising the possibility that BD-mediated suppression of Th17 responses may be mediated by microbiota alterations, potentially reflecting reduced overall microbial sensing. Together, these results suggest that BD reshapes the gut microbiota, resulting in a less diverse and more compositionally homogeneous community associated with reduced Th17 responses.

### BHB-associated Th17 suppression coincides with reduced segmented filamentous bacteria (SFB)

Differential abundance analysis using linear discriminant analysis effect size (LEfSe) identified segmented filamentous bacteria (SFB, annotated as “*Candidatus Arthromitus”*) as one of the most significantly reduced taxa in BD-treated mice (Fig. 1E). SFB are well-characterized commensals that potently induce Th17 activation through adherence to small intestinal epithelial cells and subsequent antigen presentation by dendritic cells (17, 18). Previous studies have shown that dietary components, including sugar and fiber, can modulate intestinal Th17 responses through effects on SFB abundance (19, 20). Consistent with this role, correlation analysis demonstrated that SFB was the only taxon that positively correlated with both Th17 markers assessed in the ileum in this data set (Fig. 1F). While BD treatment broadly altered the microbial community (Fig. 1E), SFB was the only taxon with a well-established and consistent association with Th17 responses. Although other taxa such as *Lactobacillus* have been linked to Th17 regulation (4), the limited taxonomic resolution of 16S rRNA sequencing precludes confident species-level interpretation. Therefore, we focused on SFB as the most robust candidate. These findings suggest that BD treatment is associated with reduced SFB abundance and suppressed Th17 responses, consistent with a model in which loss of SFB may contribute to the phenotype.

**Fig 2:**
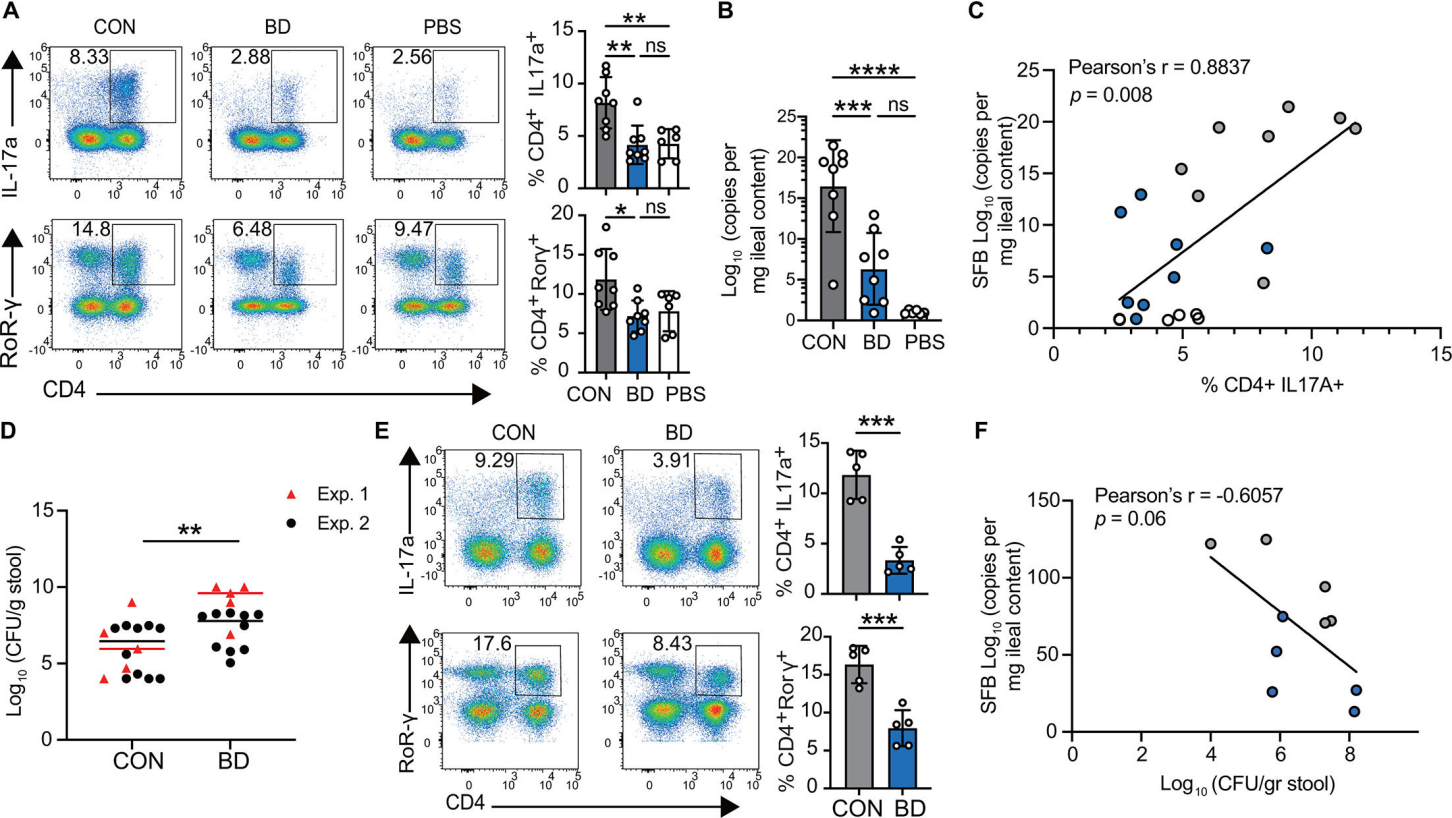
BHB alters *Citrobacter rodentium* susceptibility in association with reduced SFB abundance and Th17 responses. (**A**) Flow cytometry analysis of ileal lamina propria lymphocytes following microbiota transplantation or a PBS control. Left, representative flow plots showing CD4^+^IL-17A^+^ and CD4^+^RORγt^+^ cells gated on live CD3^+^ populations. Right, quantification of Th17 frequencies. Barplots show mean ± SD. Statistical analysis: one-way analysis of variance (ANOVA) with Tukey's multiple comparisons test. (**B**) Quantitative PCR analysis of segmented filamentous bacteria (SFB) absolute abundance in recipient mice. Bar plots show mean ± SD. Recipients of BD-shaped microbiota exhibited reduced SFB levels. Statistical analysis: one-way ANOVA with Tukey's multiple comparisons test. (**C**) Pearson’s correlation analysis showing a positive correlation between SFB abundance and ileal Th17 frequency. Colors indicate recipient groups. (**D**) Fecal *C. rodentium* burden 4 days post-infection in CON and BD-treated mice. Mice were pretreated with BD water 7 days prior to infection. Colors indicate two independent experimental repeats. Horizontal bars indicate the medians for each independent experiment. Statistical analysis: unpaired two-tailed Welch’s *t* test. (**E**) Flow cytometry quantification of ileal Th17 frequency on day 4 post-infection. Left, representative flow plots showing CD4^+^IL-17A^+^ and CD4^+^RORγt^+^ cells gated on live CD3^+^ populations. Right, quantification of Th17 frequencies. Barplots show mean ± SD. Statistical analysis: unpaired two-tailed Welch’s *t* test. (**F**) Pearson’s correlation between SFB abundance and fecal *C. rodentium* burden. *P < 0.05, **P < 0.01, and ***P < 0.001.

To determine whether the altered microbiota under BD was sufficient to recapitulate Th17 suppression, we performed microbiota transplantation experiments. Recipient mice colonized with microbiota from BD donors had significantly lower ileal Th17 frequency compared to recipients of CON microbiota (Fig. 2A). Quantitative PCR analysis revealed that absolute SFB abundance was significantly lower in recipients of BD-shaped microbiota (Fig. 2B). As observed in donor mice, SFB levels in recipients positively correlated with ileal Th17 frequency (Fig. 2C), indicating that the BD-altered microbiota is sufficient to transmit both reduced SFB levels and suppressed Th17 responses. These results support a microbiota-dependent effect of BD treatment on intestinal Th17 immunity, with reduced SFB abundance representing one potential contributing component. This observation extends that of prior work demonstrating that KD or BHB ketone ester supplementation can influence Th17 responses by increasing *Lactobacillus murinus* or reducing levels of *Bifidobacterium* under high-fat diet conditions (4, 5). Together, these studies suggest that ketogenesis may reduce Th17 responses through multiple microbiota-dependent pathways, the relative contribution of which may depend on the ecological configuration of the host microbiome. Our results add an additional layer of complexity by identifying SFB reduction as a distinct microbiota axis through which systemic BHB elevation by BD can influence Th17 levels in the intestine.

### BHB alters *C. rodentium* susceptibility in association with reduced SFB and Th17 responses

Th17 cells and their effector cytokines, including IL-17A, have been implicated in host defense against enteric pathogens such as *Salmonella* and *C. rodentium* (21). SFB colonization has been shown to confer protection against *C. rodentium* infection, in part by enhancing Th17 responses (17). To examine how BD-induced alterations in microbiota and Th17 responses relate to host susceptibility to infection, we challenged mice that had been pretreated with BD or CON water for 7 days with *C. rodentium* (22, 23). BD-pretreated mice exhibited significantly increased pathogen burden on day 4 post-infection and increased mortality compared to the CON group (Fig. 2D and Fig. S3A), although no differences in bacterial translocation to liver or spleen were observed at this time point post-infection (Fig. S3B). This corresponded with reduced intestinal Th17 levels in BD-treated mice during infection (Fig. 2E). Further, correlation analysis revealed a negative correlation between fecal *C. rodentium* load and intestinal SFB abundance (Fig. 2F). Consistent with observations in uninfected mice, BD treatment also reduced SFB abundance following *C. rodentium* infection, and SFB levels remained significantly positively correlated with Th17 markers in the ileum (Fig. S4). Together, these findings suggest that BD-mediated SFB reduction is associated with reduced Th17 responses and increased susceptibility during enteric infection.

Notably, the increased pathogen burden observed under BD coincided with reduced SFB and suppressed Th17 responses, although prior studies have demonstrated that SFB-mediated protection against *C. rodentium* during early infection can occur independently of CD4^+^ T cells (24). Thus, our findings are consistent with a model in which BD-induced BHB disrupts microbiota-associated colonization resistance and immune priming, which may contribute to increased susceptibility to enteric infection, while concurrently suppressing Th17 responses. These results extend those of prior work demonstrating that ketogenic metabolism can modulate host-pathogen interactions in a tissue-specific manner. For example, KD or BD supplementation has been shown to expand protective γδ T cells in the lung and enhance barrier function, thereby improving resistance to viral infection (10). In contrast, our data reveal in the context of an enteric pathogen that systemic BHB elevation by BD results in increased pathogen burden, suggesting an opposing tissue-specific response.

In summary, our results demonstrate that BD treatment, which leads to elevated systemic BHB, suppresses intestinal Th17 responses independent of diet composition. This phenotype is associated with remodeling of the gut microbiota, including reduced SFB abundance, and is transferable through microbiota transplantation. Of note, one limitation of the current study is that the effects of BHB versus BD were not fully parsed out due to the lack of direct BHB measurements in luminal contents and tissues. In the context of *C. rodentium* infection, BD-induced microbiota changes are linked to increased pathogen burden, highlighting a potential trade-off between altered microbiota composition and suppression of inflammatory Th17 responses in the context of host colonization resistance. Together, these findings support a diet-independent ketogenesis-microbiota-immune axis that shapes intestinal Th17 immunity and host-pathogen interactions in mice.

## Data Availability

All 16S rRNA gene sequencing data are available at the NCBI Sequence Read Archive under BioProject No. PRJNA1429443. This paper does not report original code.

## SUPPLEMENTAL MATERIAL

Supplemental Figures msystems.00324-26-s0001.docx Figures S1-S5.

Supplemental Text msystems.00324-26-s0002.docx Supplemental methods.

ASM does not own the copyrights to Supplemental Material that may be linked to, or accessed through, an article. The authors have granted ASM a non-exclusive, world-wide license to publish the Supplemental Material files. Please contact the corresponding author directly for reuse.
