# Supplementary material for: β-Hydroxybutyrate precursor 1,3-butanediol modulates enteric pathogen susceptibility and Th17 responses via commensal bacteria
Source: mSystems. 2026 Jul 16;11(8):e00324-26. doi: 10.1128/msystems.00324-26 (PMC13483238; doi:10.1128/msystems.00324-26)
Supplement: Supplementary file 1 [file msystems.00324-26-s0001.docx]

**Supplemental Information (SI)**

**Supplemental Figures**

**Fig. S1.** Gating strategy for Th17 cell populations. Cells were gated on lymphocytes, single cells, single cells, live cells, CD3+ cells, then CD4+ IL-17A+ cells or CD4+ RORγt+.

**Fig. S2.** Stacked bar plots depicting the relative abundance of bacterial taxa in control (CON) and 1,3-butanediol (BD)-treated mice at the phylum level (**A**) and genus level (**B**).

**Fig. S3**. (**A**) BD pretreatment decreased the probability of survival post *C. rodentium* infection. Data are from experiment 1 (n=5). Statistics: Logrank (Mantel-Cox) test. (**B**) Bacterial translocation to the liver and spleen 4 days post *C. rodentium* infection.

**Fig. S4.** BD reduces ileal SFB abundance, and SFB positively correlates with Th17 responses during *C. rodentium* infection. (**A**) Quantitative PCR analysis showing reduced segmented filamentous bacteria (SFB) abundance in the ileal contents of BD-treated mice 4 days after *C. rodentium* infection. Statistics: unpaired Welch’s t test. (**B**) Pearson correlation analysis demonstrating a positive correlation between ileal SFB abundance and the frequency of CD4⁺IL-17A⁺ cells gated on live CD3⁺ on day 4 post-infection.

**Fig. S5.** Body weight changes during BD treatment. Body weight was monitored throughout the experimental period and normalized to initial body weight. Data are shown as mean ± SEM. *p < 0.05, **p < 0.01; ns, not significant. Statistics: two-way ANOVA.
